# Physical Exercise and Appetite Regulation: New Insights

**DOI:** 10.3390/biom13081170

**Published:** 2023-07-27

**Authors:** Lorenzo Caruso, Enrico Zauli, Mauro Vaccarezza

**Affiliations:** 1 Department of Environmental and Prevention Sciences, University of Ferrara, 44121 Ferrara, Italy; lorenzo.caruso@unife.it; 2 Department of Translational Medicine, University of Ferrara, 44121 Ferrara, Italy; enrico.zauli@edu.unife.it; 3 Curtin Medical School and Curtin Health Innovation Research Institute (CHIRI), Curtin University, Bentley, WA 6102, Australia

**Keywords:** obesity, appetite, physical exercise, L-Lactate

## Abstract

Physical exercise is considered an important physiological intervention able to prevent cardiovascular diseases, obesity, and obesity-related cardiometabolic imbalance. Nevertheless, basic molecular mechanisms that govern the metabolic benefits of physical exercise are poorly understood. Recent data unveil new mechanisms that potentially explain the link between exercise, feeding suppression, and obesity.

## 1. Introduction

Physical activity has been demonstrated to help reduce several metabolic disorders, including obesity, diabetes, and fatty liver disease [1,2,3,4]. Despite the proven widespread advantages granted by physical exercise regarding well-being and the prevention of cardiometabolic diseases, physical activity is rather ineffective in establishing substantial weight loss. This is most likely caused by homeostatic compensatory metabolic mechanisms acting on energy intake and expenditure [5].

Exercise plays a significant role in weight control by, in part, influencing appetite regulation [6,7,8]. Engaging in a single exercise session results in a temporary energy deficit without triggering compensatory effects on appetite. Limited evidence suggests that regular exercise may alter subjective and homeostatic factors related to appetite, leading to increased feelings of fullness after meals. However, it should be noted that individuals may have varying responses to exercise in this regard [6,7,8]. Research indicates that exercise can impact energy balance by affecting energy intake levels. Therefore, several studies have investigated the relationship between exercise interventions and changes in appetite and energy intake, aiming to determine the extent to which changes in appetite contribute to exercise-induced weight management [8]. These studies have examined the effects of both single-exercise sessions and long-term exercise training programs on appetite and energy intake variables. Recently, researchers have also explored changes in appetite-related hormones to gain a better understanding of the underlying mechanisms that influence appetite and energy intake following exercise. These studies have revealed significant variability in how individuals respond to exercise in terms of appetite, appetite-related hormones, and energy intake [8]. Individual characteristics and behaviors, such as body composition, gender, and habitual physical activity, may also influence how one’s appetite responds to exercise. As appetite and energy intake are crucial aspects of energy balance, further research into the individual factors that modulate appetitive measures after exercise is vital for effective weight management strategies.

Evidence examining the acute and chronic effects of exercise on appetite, energy intake, and appetite-related hormone responses has been reviewed extensively [9,10,11,12,13,14,15]. A plethora of studies have investigated the appetite-related reactions experienced during and following individual sessions of uninterrupted aerobic exercise. A sizable portion of these studies focused on lean, physically active males. Collectively, these scientific inquiries have revealed that subjective sensations of hunger tend to diminish temporarily during exercise conducted at or above 60% of peak oxygen uptake (VO2 peak). This occurrence is commonly referred to as exercise-induced appetite suppression [8,16,17,18,19,20].

Hormones transiently secreted by endocrine cells in the digestive tract have a meal-by-meal impact on feelings of satiation and postprandial satiety. These hormones include acylated ghrelin, which stimulates appetite, and the anorexigenic signals PYY, GLP-1, and PP, which induce feelings of fullness. Research consistently shows that single sessions of aerobic exercise above the 60% VO2 peak reduce levels of acylated ghrelin in the bloodstream [19,20]. The effect of resistance exercise on acylated ghrelin is less clear, with some evidence indicating suppression and other studies showing no change in hormone levels. Similarly, while aerobic exercise has been found to increase concentrations of satiety hormones such as PYY, GLP-1, and PP, the impact appears to be less significant during resistance exercise. These hormonal fluctuations are short-lived and return to normal levels within hours after exercise.

Despite inconsistent findings, it is suggested that regular exercise can alter the sensitivity of the appetite control system by balancing the desire to eat with an improved response to satiety signals. Evidence supports this idea, showing reduced appetite and energy intake after consuming a high energy density meal in individuals participating in structured exercise training [21,22,23,24,25,26]. Leptin and insulin, two crucial regulators of the body’s nutritional state, have also been extensively studied in relation to exercise training. Leptin levels decrease after both aerobic and resistance training, while findings regarding insulin are more varied, with some studies showing a decrease after exercise training and others showing no change. The long-term effects of exercise on episodic peptides from the digestive tract remain uncertain. Some studies indicate an increase in concentrations of acylated ghrelin, PYY, GLP-1, and PP after resistance exercise training, but other evidence suggests negligible differences [21,22,23,24,25,26].

## 2. Latest Insights

According to a recently published study [27], prolonged exercise can aid in weight loss, but only if the additional calories burnt are not compensated for with increased food consumption afterwards. The authors of the article detail the discovery of a molecule released during exercise that suppresses appetite in mice. They used an ‘unbiased metabolomic approach’ to identify molecules that circulated in the blood following intense exercise in humans, racehorses, and mice. They discovered that a compound known as Lac-Phe—a conjugate of lactate and phenylalanine, which increased significantly in each species after intense exercise—was responsible for appetite suppression [27]. The researchers conducted experiments administering high doses of Lac-Phe to diet-induced obese mice, which decreased their food intake and body weight. However, this effect was not observed in lean mice on a low-fat diet [27]. The authors confirmed that carnosine dipeptidase 2 (CNDP2) produces Lac-Phe and that lactate plays a role in its production, which is consistent with its generation during exercise. These findings offer insight into the physiological effects of exercise and offer a potential avenue for future research.

The absence of CNDP2 in genetically engineered diet-induced obese mice resulted in a failure to upregulate Lac-Phe production in response to exercise. It is noteworthy that sedentary mutant animals did not exhibit significant differences in food intake and body weight compared to the controls, indicating that CNDP2 is not essential for basal energy maintenance. Li et al. conducted a study on three groups of mice: one group was fed a high-fat diet and was subjected to daily exercise; a control group of animals maintained a stable weight; and CNDP2-deficient mice displayed an increase in appetite and weight gain. The findings indicate that the CNDP2-mediated production of Lac-Phe promotes weight loss in mice fed high-fat food [27]. Although the physiological pathway through which Lac-Phe acts is unknown, current research shows that this metabolite circulates in the blood for less than one hour after exercise, yet it has long-lasting effects on the signaling pathways that alter feeding behavior. Identifying the Lac-Phe receptor protein and the cell types expressing this receptor is necessary for further investigation into the potential interaction of Lac-Phe with neurons controlling eating behaviors. An interesting possibility is that the putative receptor of the Lac-Phe complex is part of the orphan G-coupled receptor (GPCR) family [28,29], and GCPRs that recognize small peptides could be the relevant molecules that transmit signals inside CNS cells involved in hunger and food intake regulation. Studies in animal models should encompass several experiments to characterize the pathways and the CNS regions that are involved—coupling tissue/cell-specific/knockout gene experiments, electrophysiology assessment, and Lac-Phe microinjection in various brain regions should be pivotal in better understanding Lac-Phe physiology.

Neural circuits have a considerable influence on appetite through various processes such as the energy-sensing system, physiological stressors, and reward–drive pathways [30,31,32]. The presented data suggest that the modulation of any of these pathways can affect appetite. Lac-Phe has been observed to reduce appetite exclusively in mice that are fed a high-fat diet. While evidence indicates that this molecule may have a direct effect on pleasure-driven eating, further research is needed to confirm this. Exercise produces additional molecules, such as growth differentiation factor 15 (GDF15), peptide tyrosine–tyrosine, and glucagon-like peptide-1, which inhibit food intake and affect metabolic functions such as energy expenditure, insulin sensitivity, and blood glucose levels [31,32]. The chronic administration of Lac-Phe was found to improve glucose uptake in DIO mice. While the cause was originally thought to be weight loss, future studies should aim to establish whether Lac-Phe directly affects glucose uptake by tissues. Such results would reveal additional roles for these metabolites beyond curbing appetite, such as potentially affecting nutrient utilization during exercise. 

Several clinical studies have suggested that post-workout appetite suppression occurs in men, but there is limited evidence to support this hypothesis in women. The study conducted by Li et al. solely looked at male mice. Future studies should include female subjects and investigate Lac-Phe activity in this population. The different hormonal backgrounds as different hormonal and intertwined metabolic regulation between male and female individuals play a role in these contradictory preliminary findings. It is also important to examine the correlation between Lac-Phe levels in blood plasma while working out and appetite suppression and weight loss. Could the levels of Lac-Phe in plasma impact the ability of exercise to induce weight loss? The evolutionarily beneficial aspect of exercise-induced appetite suppression is intriguing. One explanation is that the strenuous exertion examined by Li et al. denotes a stressor that endangers immediate survival. Energy intake becomes a low priority because it is not necessary for short-term survival. For instance, it is better not to focus on hunger while attempting to escape predators. The study of Li et al. provides a starting point from which to examine molecular pathways for exercise-induced weight loss in obese mice. Moreover, the elevation in plasma Lac-Phe seen in men after exercising points towards opportunities for improving weight management if the role of Lac-Phe in appetite and body weight regulation translates into humans. In this regard, recent work in humans (in vitro and in vivo, mimicking exercise conditions in muscle cells [33,34]) shows that acute exercise increases plasma Lac-Phe levels. Higher Lac-Phe plasma levels were related to a greater reduction in abdominal subcutaneous fat and to a smaller reduction in visceral adipose tissue during an eight-week supervised training intervention [34]. These results suggest that Lac-Phe could have a dual role, for example in food intake, potentially via GPCRs in the CNS, as well as on adipose and other tissues. The transient repression of hunger mediated in the CNS could help to maintain blood flow in the skeletal muscle, and it has been demonstrated that lactate levels are correlated with hunger repression [35,36] and that exogenous lactate administration has appetite-suppressive effects [35,36]. With lactate being a driver of Lac-Phe production, we could hypothesize that Lac-Phe is the main player in the appetite-suppressing lactate pathway. As reported in the published literature, future studies are needed to confirm and extend these findings in humans as well as to further assess the function of Lac-Phe as a potential ‘exerkine’.

Various elements such as communal eating and the easy availability of delectable high-calorie food can impede the success of any single approach. As a result, utilizing multiple interventions simultaneously will prove to be the most effective method of addressing obesity, regardless of whether the current outcomes can be applied to humans.

Other recently published findings [32,37,38,39] focus on a systems biology approach to determine the molecular determinants of the effects of physical exercise. The Molecular transducers of Physical Activity Consortium (MoTrPAC) paper [37] illustrates a multi-omics and bioinformatics analysis that will lead to a molecular map of exercise. This project will undoubtedly shed light on exercise-induced pathways and on the intertwined connections between metabolism regulation, appetite, and physical activity itself. Moreover, a new genome-wide association analysis combining data from more than 700,000 individuals from 51 previous studies in a multi-ancestry meta-analysis [38], on moderate to vigorous physical engagement during leisure time, leisure screen time, and sedentary behavior yielded 104 new independent association signals in 99 genetic loci. It is not unexpected that intricate mechanisms of behavior, encompassing both physical activity and motivation, manifest in multiple organs and systems, with the brain and muscles playing crucial roles. Further analyses will enhance our comprehension of the molecular foundations behind leisure time physical activity, appetite regulation, and the diverse molecular impacts of leisure and exercise on appetite behavior. Lastly, new data show the role of growth differentiation factor 15 (GDF15) in appetite control and physical exercise, influencing appetite regulation and counteracting compensatory reductions ion in energy expenditure [32,39]. GDF15 serves as a widespread cellular stress indicator that travels through the bloodstream to reach the brainstem. Once there, it interacts with the glial cell-derived neurotrophic factor family receptor alpha-like (GFRAL-RET), triggering a series of hormonal and behavioral reactions, one of which is a decrease in food consumption. In certain pathological circumstances such as cancer, sepsis/infection, and mitochondrial disease, as well as during extended physical activity and pregnancy, the levels of GDF15 in the bloodstream are notably heightened. Given the significant and diverse health advantages associated with exercise, it is important to delve deeper into this specific aspect of GDF15′s function [32]. GDF15 has been demonstrated very recently to stimulate beta adrenergic activity and skeletal muscle calcium cycling [39]. The preservation of energy expenditure in GDF15-treated mice, in contrast to calorically restricted pair-fed controls, is linked to elevated levels of noradrenaline in skeletal muscle, increased fatty acid oxidation, higher oxygen consumption, and an upsurge in futile calcium cycling [39]. The observed effects of GDF15 on stimulating thermogenesis in skeletal muscle could potentially elucidate the reasons behind the observed increase in weight loss when combined with incretin-based therapeutics, known for their ability to suppress appetite [32]. It is conceivable that Lac-Phe secretion could exert its activities together with the GDF15 axis and a synergic action cannot yet be ruled out. These new avenues open new perspectives on the various molecular mechanisms of the effects of physical exercise, allowing a deeper understanding of exercise benefits and improving our potential delivery of exercise as an effective ‘drug’ in various settings and combinations.

## 3. Conclusions and Future Directions

Current evidence suggests that physically active individuals have improved appetite sensitivity, which could generate long-term energy balance. Thus, exercise should be promoted as a universal method of inducing an acute energy deficit, and engaging in regular physical activity may promote a closer link between energy intake and energy expenditure in the longer term.

Further research is essential to gain a comprehensive understanding of how adiposity, sex, and habitual physical activity impact appetite, energy intake, and the response of appetite-related hormones to both acute and chronic aerobic and resistance exercise. Additionally, it is important to explore various inter-individual physiological factors in relation to exercise and appetite parameters.

One area of investigation should focus on the influence of genetic polymorphisms associated with body adiposity on appetite-related responses to exercise. Specifically, variations in genes related to obesity and appetite should be studied to determine how they affect appetite, energy intake, and hormone responses during exercise. Of particular interest are single-nucleotide variations in the fat mass and obesity-associated gene, as carriers of ‘obesity-risk’ variants tend to experience heightened postprandial appetite, increased energy intake, and higher concentrations of acylated ghrelin. Therefore, future experiments should explore whether exercise can offset the appetite and body mass differences observed in individuals carrying different variants of this gene.

Additionally, more research is needed to investigate the impact of varying levels of fat-free mass on post-exercise appetite and appetite-related hormones. It is crucial to examine how exercise modality and changes in body composition interact to influence appetite-related measures, especially considering that resistance training leads to greater increases in fat-free mass compared to traditional aerobic training. By comparing groups with distinct levels of adiposity and fat-free mass, we can enhance our understanding of how different body tissues contribute to appetite regulation and energy balance.

Furthermore, it is necessary to examine the effects of acute and chronic exercise on appetite and appetite-related hormones across a range of ages. Weight gain commonly occurs during adulthood, followed by weight loss starting from the age of 65 years and beyond. While metabolic and musculoskeletal mechanisms are believed to be involved, changes in appetite also play a role. This becomes particularly relevant in older individuals as they often experience a decline in energy intake, which is potentially linked to increased levels of leptin and cholecystokinin, and a decrease in ghrelin.

In conclusion, conducting further studies that consider the impact of adiposity, sex, and habitual physical activity on appetite, energy intake, and appetite-related hormone responses to exercise is pivotal. Exploring genetic variations, changes in fat-free mass, and age-related effects would contribute to a better understanding of the complex relationship between exercise and appetite regulation, improving our knowledge of energy balance.

The most recent insights into the molecular pathways involved in exercise-induced weight loss are the starting point for future improvements in weight management if the role of Lac-Phe in appetite and body weight modulation demonstrates translation into the clinic. It is conceivable that several factors, such as social eating and access to high-calorie palatable food, can bypass the effectiveness of any ‘standalone’ interventions. Multiple strategies used together may be the best way to combat obesity.
